# Banking or Bankrupting: Strategies for Sustaining the Economic Future of Public Cord Blood Banks

**DOI:** 10.1371/journal.pone.0143440

**Published:** 2015-12-01

**Authors:** Jeremy Magalon, Martin Maiers, Joanne Kurtzberg, Cristina Navarrete, Pablo Rubinstein, Colin Brown, Catherine Schramm, Jérome Larghero, Sandrine Katsahian, Christian Chabannon, Christophe Picard, Alexander Platz, Alexander Schmidt, Gregory Katz

**Affiliations:** 1 ESSEC Business School, Paris-Singapore, France; 2 Assistance Publique Hôpitaux de Marseille CIC BT 1409, Marseille, France; 3 Vascular Research Center of Marseille, Aix Marseille University, Marseille, France; 4 National Marrow Donor Program, Minneapolis, Minnesota, United States of America; 5 Carolinas Cord Blood Bank, Duke University, Durham, North Carolina, United States of America; 6 NHS Cord Blood Bank, NHS Blood and Transplant, London, United Kingdom; 7 National Cord Blood Program, New York Blood Center, New York, New York, United States of America; 8 INSERM U1138, Paris Descartes University, UPMC, Paris, France; 9 Assistance Publique-Hôpitaux de Paris, Paris, France; 10 Institut Paoli-Calmettes, CIC BT 1409, Marseille, France; 11 EFS Alpes Méditerranée, Marseille, France; 12 DKMS Cord Blood Bank, Dresden, Germany; Universidade de São Paulo, BRAZIL

## Abstract

**Background:**

Cord blood is an important source of stem cells. However, nearly 90% of public cord blood banks have declared that they are struggling to maintain their financial sustainability and avoid bankruptcy. The objective of this study is to evaluate how characteristics of cord blood units influence their utilization, then use this information to model the economic viability and therapeutic value of different banking strategies.

**Methods:**

Retrospective analysis of cord blood data registered between January 1st, 2009 and December 31st, 2011 in Bone Marrow Donor Worldwide. Data were collected from four public banks in France, Germany and the USA. Samples were eligible for inclusion in the analysis if data on cord blood and maternal HLA typing and biological characteristics after processing were available (total nucleated and CD34+ cell counts). 9,396 banked cord blood units were analyzed, of which 5,815 were Caucasian in origin. A multivariate logistic regression model assessed the influence of three parameters on the CBU utilization rate: ethnic background, total nucleated and CD34+ cell counts. From this model, we elaborated a Utilization Score reflecting the probability of transplantation for each cord blood unit. We stratified three Utilization Score thresholds representing four different banking strategies, from the least selective (scenario A) to the most selective (scenario D). We measured the cost-effectiveness ratio for each strategy by comparing performance in terms of number of transplanted cord blood units and level of financial deficit.

**Results:**

When comparing inputs and outputs over three years, Scenario A represented the most extreme case as it delivered the highest therapeutic value for patients (284 CBUs transplanted) along with the highest financial deficit (USD 5.89 million). We found that scenario C resulted in 219 CBUs transplanted with a limited deficit (USD 0.98 million) that charities and public health could realistically finance over the long term. We also found that using a pre-freezing level of 18 x 10^8^ TNC would be the most cost-effective strategy for a public bank.

**Conclusion:**

Our study shows that a swift transition from strategy A to C can play a vital role in preventing public cord blood banks worldwide from collapsing.

## Introduction

As cord blood is an important source of stem cells, cord blood banks can play a key role in the industrial future of regenerative medicine [1,2]. However, a survey conducted by the World Marrow Donor Association (WMDA) revealed that only 16 of 139 public cord blood banks operating worldwide in 2013 were financially sustainable [3]. In a context of economic austerity entailing a downturn in public and philanthropic funding, public banks are struggling to break even. Moreover, WMDA reports show that the growth in the number of cord blood units (CBUs) transplanted is leveling out [4], probably due to the emergence of haploidentical transplantation which represents an alternative to cord blood transplantation [5]. In 2014, France decided to shut down half of its operating public banks just two years after launching a national development plan to support public banking. Without new banking strategies, many more public banks are likely to close in the coming years [6].The economic vulnerability of public banks requires that selective criteria be established to help decide which CBUs are eligible for cryopreservation. Composite scoring systems have been proposed to measure the biological quality of collected units before storage and after thawing to help transplant physicians obtain the best possible grafts for their patients [7]. The parameters making up these scores directly impact costs and prices according to the banks’ selectivity levels [8].

Each transplanted CBU represents a life-saving opportunity for patients, as well as a source of revenue and financial stability for banks. The economic viability of allogeneic unrelated cord blood banks is mainly dependent on the utilization rate of CBUs. The utilization rate is defined as the number of transplanted CBUs divided by the overall number of banked CBUs over a given time period. To maximize the utilization rate (output) and minimize bank operating costs (input), public banks are adopting new strategies for selecting CBUs based on a range of quality criteria. Apart from Human Leukocyte Antigen (HLA), the number of total nucleated cells (TNCs) is recognized as the main criterion used by transplant centers for selecting CBUs from public banks [9–13]. In terms of HLA compatibility, the ethnic profile of the CBU is assumed to have an impact on the utilization rate. Studies have shown that ethnic minorities that are underrepresented in public registries are overexposed to the risk of not finding a compatible graft [14–16]. However the level of influence of the ethnicity factor on the UR has not been assessed in the literature mainly because, in many countries, the ethnic profile distribution of stored CBUs is not available.

The primary aim of this study is to evaluate how the biological characteristics of CBUs (cell counts and ethnicity) influence their utilization. Using the criteria identified as influencing the utilization rate, the viability of different banking strategies is evaluated to identify a sustainable operating model for public blood banks.

## Methods

This study analyzed a sample of CBUs registered in Bone Marrow Donor Worldwide (BMDW) between January 1, 2009 and December 31, 2011. In total, 9,396 CBUs met the inclusion criteria for the study (Table 1). Samples were supplied by four public banks based in France (Paris and Marseille, Réseau Français de Sang Placentaire, RFSP), the United States (Durham, North Carolina, National Marrow Donor Program, NMDP) and Germany (Dresden cord blood bank, DKMS). The distribution of CBUs for each bank is presented in a supporting information file (S1 Table) as well as the characteristics of CBUs (S2 Table). The cord blood banks in Paris and Marseille are WMDA and JACIE accredited; the bank in Durham is accredited by FACT-NETCORD; and the bank in Dresden is FACT-NETCORD and WMDA accredited. Although different in many respects, these banks operate in a homogenous fashion as they systematically perform maternal HLA typing for each cryopreserved CBU. Transplantations were analyzed until June 30^th^, 2013.

**Table 1:** CBU inclusion criteria.

**CBUs Registration in the BMDW registry**	Between January 1^st^,2009 and December 31^st^, 2011
**Electronic HLA typing**	For the CBU: at least generic HLA-A, -B, and allelic HLA-DRB1 typing
	For the mother: at least generic HLA-A, -B typing
**Biological characteristics**	Total Nucleated Cell count after processing
	CD34+ cell count after processing
**CBUs Transplantation**	From January 1^st^, 2009 through June 30^th^, 2013

For each CBU included in the sample, at least generic (HLA-A, -B) and allelic (HLA-DRB1) genotyping were performed. For maternal samples, only generic genotyping was performed. In order to correlate the CBUs’ HLA haplotypes with the parents’ ethnic background, we used the NMDP database built on existing high resolution HLA typing [17]. This database refers to the classification of individuals defined by the utilization score census based on four ethnic origins: Caucasians, Asians, Africans, and Hispanics. For each CBU analyzed in our sample, both parental haplotypes were inferred by comparing the CBU’s HLA profile with the mother’s partial HLA. According to the NMDP database, and based on published results [18,19], we used a standard protocol to deduce the geographic origin of both parents according to demographic frequencies and the distribution of ethnic backgrounds [20].

Two groups of CBUs were distinguished: (i) a “Caucasian” group in which both parents transmitted HLA haplotypes featuring Caucasian HLA types; (ii) a “Non-Caucasian” group in which at least one HLA haplotype was a non-Caucasian marker. This distinction was fully automated using the R statistical software.

The automated algorithm allowed for large-scale assessment as to whether or not a CBU collected from a newborn with at least one non-Caucasian haplotype (a non-Caucasian CBU) had a higher probability of being transplanted than a Caucasian CBU. Key CBU characteristics were assessed for differences between groups (Caucasian and non-Caucasian) using a t-test, whereas the utilization rate for CBUs from Caucasian and non-Caucasian groups was compared using the Chi-squared test. A multivariate logistic regression model was used to assess the level of influence of three parameters—ethnic background, TNC and CD34+ cell counts—on the CBU’s utilization rate. To account for potential variation between sample sources, the model integrated a level of heterogeneity through the addition of a random cord blood bank effect. In the model, this randomization facilitated treating the four banks as if they were integrated into a single cord blood registry operating across three countries (France, Germany, and the United States).

Among the three parameters, we select those providing the model with the lowest Akaike Information Criterion value (AIC measures the relative quality of a statistical model for a given set of data). From this model, we derived a formula reflecting the probability of utilization, defined as the CBU’s Utilization Score (Eq 1). The Utilization Score has TNC count and CD34+ cell count as its inputs. The ability of the Utilization Score to discriminate between utilized and non-utilized CBUs was estimated using the receiver operating characteristic (ROC) curve [21,22]. The area under the ROC curve (AUC) quantifies the discrimination of the Utilization Score, with an AUC of 1.0 indicating perfect discrimination. As sensitivity (identification of true positives) and specificity (true negatives) have an inverse relationship that is dependent on the degree of overlap within their distributions, the optimal Utilization Score threshold corresponds to the point on the ROC curve that maximizes the sum of sensitivity and specificity. All statistical analyses were performed with the R software package, and all statistical tests were two-sided with significance tested at the 5% level.

$$\text{CBU Utilization Score}=\;\frac{\text{exp}(-6.736+0.192\text{X}+0.040\text{Y})}{1+\;\text{exp}(-6.736+0.192\text{X}+0.040\text{Y})}$$ Eq 1

where X is the TNC count (x10^8^) and Y is CD34+ count (x10^6^).

The Utilization Score was used to inform four modeled banking strategies, with the Utilization Score threshold reflecting the selectiveness with which the bank’s CBU recruitment choices were made. The four strategies considered were: (A) free recruitment with no consideration for the Utilization Score; (B) selective recruitment of Utilization Score >0.0089; (C) selective recruitment of Utilization Score >0.0308; or (D) selective recruitment of Utilization Score >0.0776. Model outcomes were defined as the number of transplanted CBUs (therapeutic value) and the bank’s profit (economic value). For each CBU in the model, if its Utilization Score is greater than the threshold, the probability of the CBU being utilized for transplant is equal to the sensitivity associated with the threshold; otherwise the probability that the CBU is not transplanted is equal to the specificity associated with the threshold.

Recruitment and processing costs as well as distribution revenues were integrated into the model based on published data reported by the NMDP and the Swiss registries [23]. The study published in 2013 by Bart *et al*. refers to data provided by banks operating in continental Europe and the USA, which is similar to our study. The operational costs and prices were considered relevant and were utilized to our analysis. This includes cost of supplies, equipment and labor associated with the recruitment, collection, transportation, processing and storage of CBUs for public use. When all costs were taken into account, the average cost of each unit actually stored was USD 1,524 including indirect expenses for units that were collected but not banked due to a variety of reasons (67%), including failure of testing, below bankable size, etc. It was assumed that only 33% of recruited units were processed and banked (average selectivity). According to Bart *et al*. direct cost to recruit was estimated at USD 206; direct cost to recruit and process at USD 1,092; net profit per distributed CBU at USD 29,237. The economic impact of each scenario was evaluated to identify the most likely scenario to be cost effective with respect to CBUs transplanted.

## Results

Of the 9,396 samples, Caucasians and non-Caucasians accounted for 5,815 and 3,581 samples, respectively (Table 2). A between group univariate analysis found that there was no significant difference with respect to TNC count (p = 0.48) or the CBU utilization rate (p = 0.37). Differences were observed in the level of CD34+ after processing, with the Caucasian group having significantly higher values compared with the non-Caucasian group (p<0.0001). Further differences were identified if only those CBUs that were transplanted were analyzed. In this case, the levels of TNC (p<0.0001) and CD34+ (p = 0.0008) were both significantly higher for the Caucasian group.

**Table 2:** Biological differences between the “Caucasian” and “Non-Caucasian” groups.

Group	Caucasian	Non-Caucasian	p value
**OVERALL SAMPLE OF CBUs**			
Number of CBUs	5,815	3,581	-
Total Nucleated Cells (x10^8^) (± SD)	14.2 (± 5.49)	14.3 (± 5.08)	0.48
CD34+ cells (x10^6^) (± SD)	5.2 (± 6.9)	4.2 (± 3.9)	< 0.0001
CBUs released for transplantation	168	116	-
Utilization Rate (%)	2.89	3.24	0.37
**SAMPLE OF TRANSPLANTED CBUs**			
Total Nucleated Cells (x10^8^) (± SD)	24.06 (± 6.2)	20.5 (± 6.26)	< 0.0001
CD34+ (x10^6^) (± SD)	9.25 (± 3.61)	7.33 (± 5.96)	0.0008

In the multivariate analysis, both the levels of TNC and CD34+ significantly influenced the utilization rate (Table 3). The derived model indicated that each additional unit of TNC (1 x 10^8^) and CD34+ (1 x 10^6^) was associated with an odds ratio of 1.212 (95% confidence interval 1.186–1.238) and 1.041 (95% confidence interval 1.021–1.061), respectively, for increased utilization. As both variables are cell counts, levels of TNC and CD34+ could be correlated and the relative influence of the two factors in the model should not be interpreted as TNC having a larger influence than CD34+. A series of univariate analyses, however, demonstrated that the model had better discriminative power when both variables were included compared with only one of the two.

**Table 3:** Multivariate analysis to assess the comparative influence of ethnic background, TNC and CD34+ counts on the UR.

	Odds Ratio	Lower Confidence Interval	Upper Confidence Interval	p value
Ethnic Background	0.996	0.765	1.296	0.97
TNC (x10^8^)	1.212	1.186	1.238	< 0.0001
CD34+ cells (x10^6^)	1.041	1.021	1.061	< 0.0001

**Fig 1:**
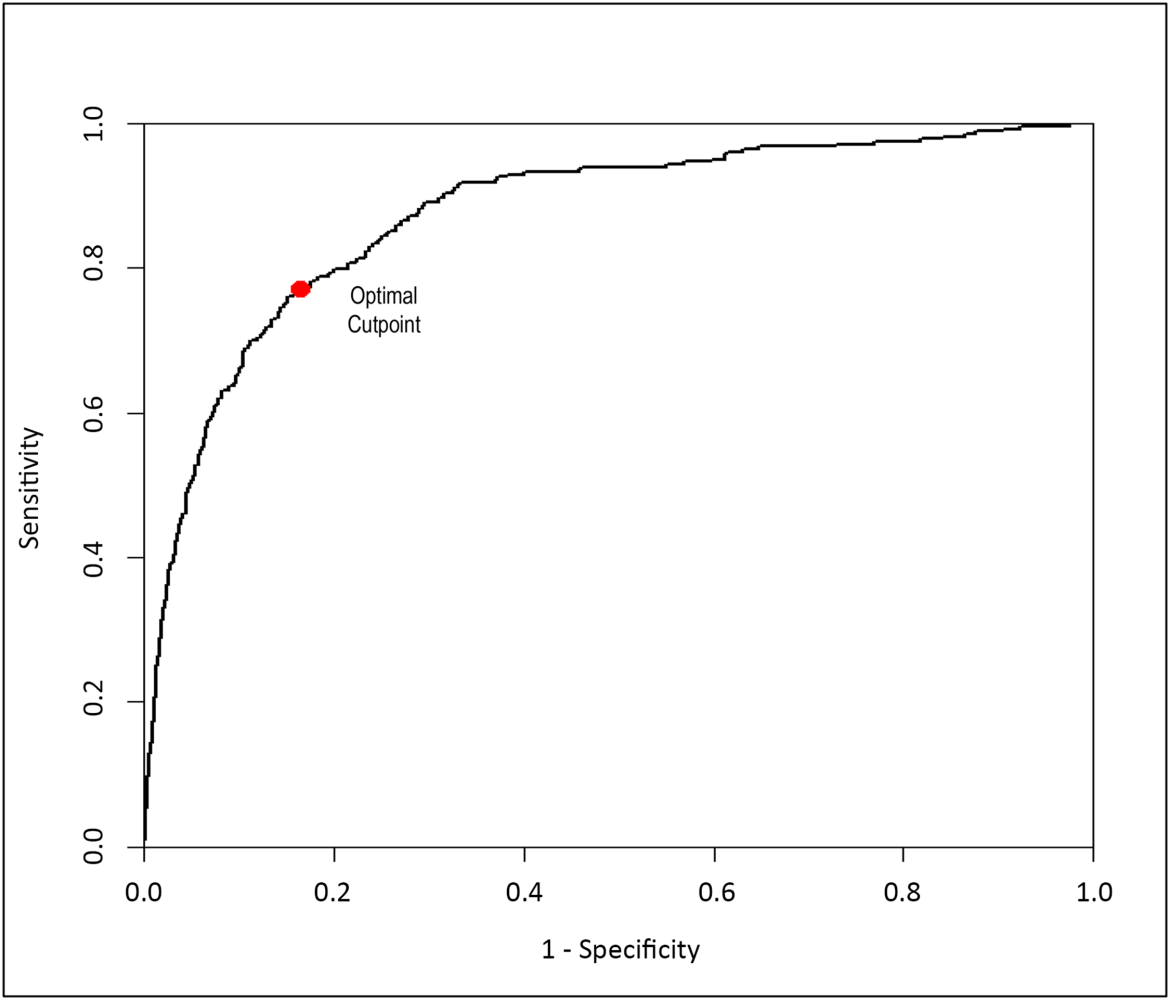
ROC curve derived from multivariate analysis.

**Fig 2:**
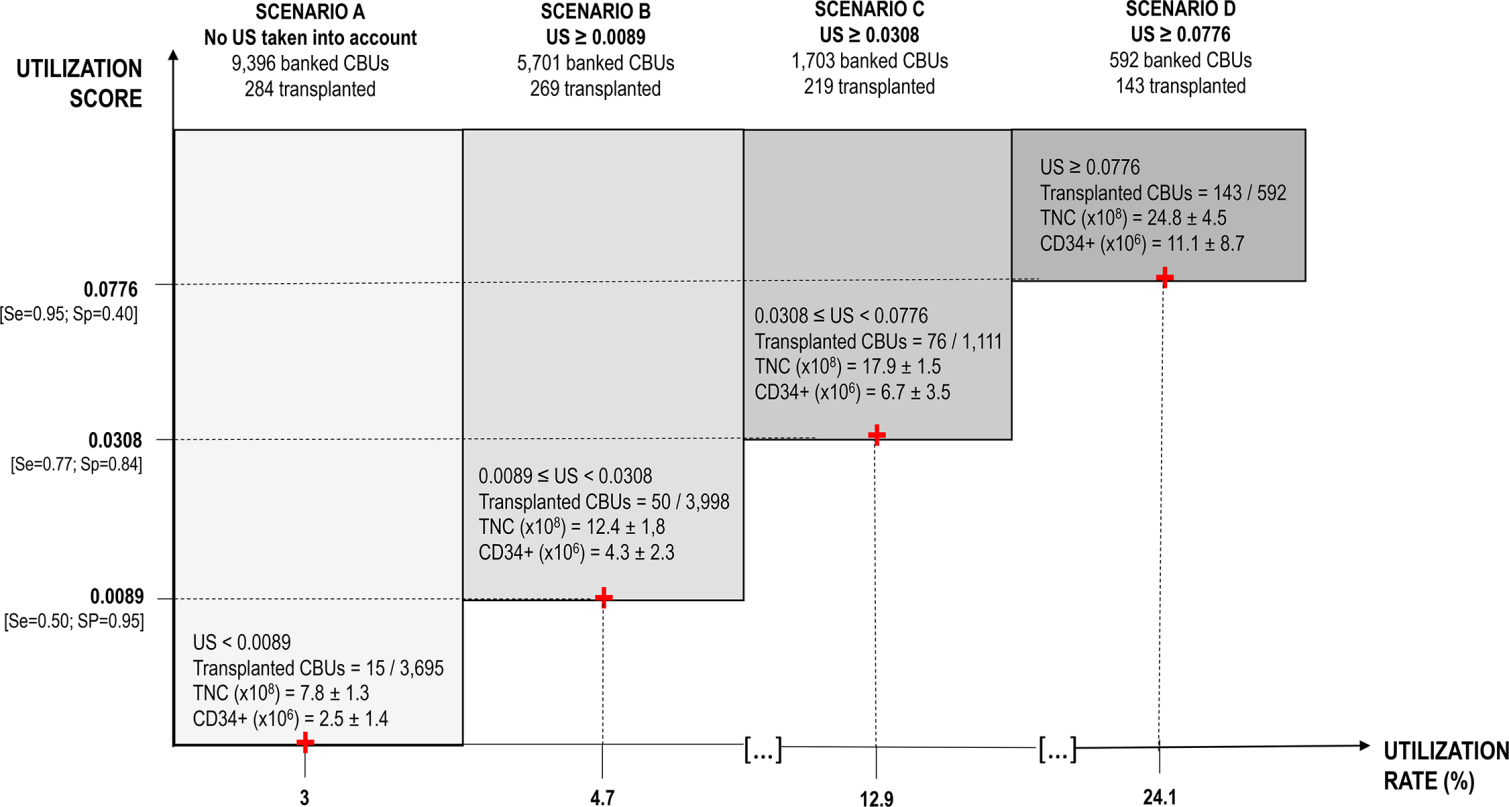
Banking strategies according to utilization rate and utilization score (US).

The multivariate analysis was used to inform the Utilization Score. ROC analysis was performed including and excluding data on ethnicity, with the AUC being 0.8754 in both cases (Fig 1). This provided further evidence that the ethnic background did not influence the CBU utilization rate. The maximal sum of sensitivity and specificity was found at a CBU Utilization Score of 0.0308. The value of 0.0308 is used as the threshold for Scenario C when modeling the different banking strategies (Fig 2).

**Fig 3:**
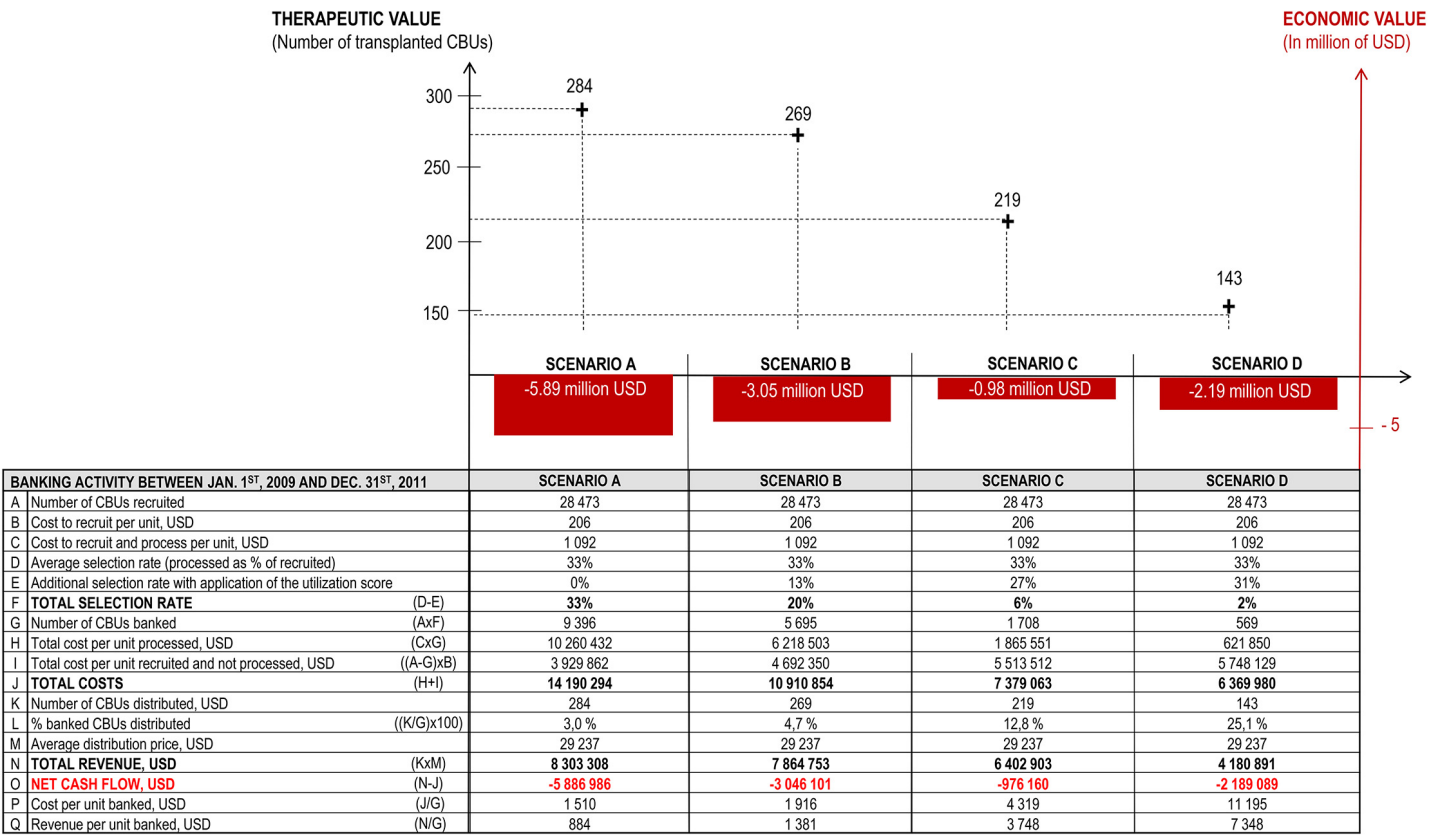
Therapeutic and economic value based on banking strategies. Four recruitment strategies were considered: (A) Banks implement an average selectivity rate of 33%, which reflects the percentage of banked units vs. collected units. The level is increased by applying the Utilization Score up to a total selectivity of 20% (B), then 6% (C) and 2% (D). For each scenario, therapeutic value (left scale) is indicated by the number of transplanted CBUs and confronted to the economic value in USD (right scale). The table presents the operating results for each scenario.

In modeling the viability of the four different banking strategies, there were substantial differences apparent between the most tolerant strategy (Scenario A) and the selective strategies (Scenarios B, C and D). Based on the study sample (9,396 CBUs banked from 2009 to 2011), a group of banks adopting Scenario A would incur a deficit of USD 5.89 million. A deficit was also apparent in Scenario B and D, where costs were higher than revenues by approximately USD 3.05 million and USD 2.19, respectively. Under Scenarios C, the cord blood bank deficit becomes significantly more limited (USD 0.98 million). As the choice between these strategies in the real-world is likely to have significant economic implications for the future of public banks, scenarios A, B and D would be non-viable. Scenarios C resulted in a limited deficit that charities, fund raising and/or public grants could realistically finance over the long-term (Fig 3). Scenario C is the most cost-effective when both the economic bottom line and the therapeutic value delivered to patients are considered together.

Analysis of model simulation results identified that more selective banking strategies resulted in a higher mean cost per CBU banked. The mean cost of banking one CBU in Scenario D (USD 11,195) was nearly 8 times higher than in Scenario A (USD 1,510). Likewise, the mean revenue per CBU banked increased, the value in Scenario D (USD 7,348) being over 8 times higher than in Scenario A (USD 884). Results suggest that selective banking strategies making use of the Utilization Score developed in this work are most likely to be financially sustainable for public banks.

## Discussion

Assessing the optimal size of a public cord blood bank represent a health economic challenge. In 2010, Querol *et al*. proposed a minimal pre freezing level of 9 x10^8^ TNC for the UK public banks to provide 98% of the population in need with a 4/6 HLA match level [24]. However, because in 2013 only 16 public banks out of 139 declared being financially sustainable, the challenge is not only to *provide—*but rather to *afford to provide* compatible units for everyone. Based on the economic risk of bankruptcy that public banks are facing, our study combines both therapeutic and economic values through four banking strategies. We found that using a pre freezing level of 18 x 10^8^ TNC would be the best strategy that delivers the highest therapeutic value to patients with the lowest financial deficit for the bank.

In order to drive costs down and utilization rates up, public banks need to continue to invest in training obstetricians and mid-wives to improve collection performance. They also need to optimize donor selection based on maternal and infant characteristics that influence CBU quality [25].

Literature reports that donor’s ethnic profile is an essential factor in providing a high level of HLA compatibility to minorities [14,16]. A study conducted in Marseille (France)—a highly ethnically diverse metropolitan area—revealed that, when a public bank collects CBUs from a maternity center welcoming ethnic minorities, this significantly increases the bank’s HLA diversity compared to the regional bone marrow registry and the global registry of CBUs in BMDW [18]. Such a maternity-based recruitment strategy would provide rare and diverse haplotypes which may be able to benefit a greater number of patients. This would imply a higher utilization rate for CBUs with a non-Caucasian ethnic background. However, this hypothesis was difficult to verify in many countries due to a restricted access to data related to the donor’s ethnic background.

This study overcame this difficulty by creating an algorithm combining CBUs and maternal HLA profiles, with an existing database correlating haplotypes with ethnic origins. Applied homogeneously to a large cohort, this automated tool revealed that CBUs collected from newborns with at least one non Caucasian parent have a utilization rate equivalent to those with two Caucasian parents.

It should be underlined that the Caucasian group represented 62% of our sample. With only 38%, the non-Caucasian group aggregates three other distinct ethnic sub-groups (Hispanic, African, and Asian) as well as mixed-race individuals with up to one Caucasian haplotype. The fact that the non-Caucasian group was both smaller and more diverse compared with the Caucasian group may have created a bias, and makes it difficult to conclude to what extent ethnicity influences the utilization rate of CBUs. Indeed, studies show that transplant physicians often preferentially select lesser matched CBUs with a high cell count for transplant in patients with minority backgrounds [8]. In this study we were unable to measure the number of Caucasian CBUs transplanted to non-Caucasian patients and vice-versa. The recruitment of CBUs from non-Caucasian donors remains a priority in order to offer ethnic minorities a graft with an equivalent level of compatibility to that generally found for Caucasians, who are over-represented in registries worldwide.

It should also be noted that our analysis focused on utilization, deliberately omitting any information about clinical outcomes of the transplantation. Multivariate analysis of CBU characteristics influencing the utilization rate permitted the elaboration of a Utilization Score that estimated the probability for each CBU to be transplanted. Based on different hypotheses in terms of specificity and sensitivity, scenarios were modelled to represent different banking strategies.

As transplantation represents a clinical benefit for the patient, the number of transplanted CBUs produced by the bank was assumed to be a measure of therapeutic value. The financial outcome of each scenario measured the bank’s economic value, i.e. its capacity to balance costs and revenues and secure its operational viability. When comparing inputs and outputs for the four scenarios, Scenario A represented the most extreme case as it delivered the highest therapeutic value for patients (284 CBUs transplanted) along with the highest financial deficit (USD 5.89 million). A non-selective banking strategy is, therefore, financially unsustainable.

Although the overarching goal of a public bank is not to break-even, an unsustainable financial banking model will certainly fail to deliver therapeutic value to patients over the long term. As such, economic stability is required for therapeutic benefit to be realized. This is the case of Scenario C which resulted in a significant therapeutic benefit delivered with an economic deficit affordable for public health.

The case of the French national network of public banks (RFSP) is illustrative in the context of this discussion, as its development plan showed a radical strategic shift from scenario A to C within just a few years [26]. In 2006, France ranked only 15^th^ in terms of cryopreserved CBUs per inhabitant [27]. In 2008, the RFSP invested public money in a development plan to expand its network of banks and maternity centers in order to rapidly increase the quantity of CBUs with a minimal TNC threshold after processing of 6 x 10^8^ (which would fall within Scenario A). This threshold increased to 10 x 10^8^ in 2013 (approximating to Scenario B). By the end of 2013, a drastic shortage of public funding forced six public banks to close down their activities only three years after launching. To avoid bankruptcy in 2014, the remaining operating banks decided to radically change their recruitment strategy and increase the TNC threshold to 16 x 10^8^ (approximating to Scenario C). Within seven years, the cell dose criterion after processing has increased by 267%. In a context of economic austerity, the rapid transition from strategy A to C turns out to be vital in order to prevent the network of public banks from collapsing.

Our study provides a new tool for implementing this strategy shift. With the annual storage cost of a CBU evaluated at USD 27 [23], our model may also help public banks screen and, if required, potentially discard cryopreserved CBUs with a very low probability of being utilized. Although this would represent significant cost savings for the bank, this option has to be considered in conjunction with the potential for future cell expansion techniques [28, 29]. Still in development, expansion protocols may increase the quantity of CD34+ and, as a result, the utilization rate of cryopreserved CBUs. However, cell expansion will also come with a cost, further impacting the development strategies of public banks. In addition, the generation of induced pluripotent stem cells (iPS) from small number of cryopreserved CBUs may serve as a tool to study disease mechanisms, and could eventually lead to the development of iPS-derived therapies [30]. In order to make regenerative therapies available to the population at large, the development of allogeneic, off-the-shelf products will be necessary for the next decade [31].

Other strategies to select CBUs are evolving with the use of HLA-C [32] as well as the identification of non-inherited maternal antigen (NIMA) [33]. These two approaches challenge the idea that only CBUs with the highest levels of TNC are the more likely to be utilized. However, our analysis does not take into account NIMA, HLA-C typing or the cost of high resolution typing because these technologies were not implemented by the four banks during the inclusion period from 2009 to 2011.

## Conclusion

Our study shows that the utilization rate of CBUs is paramount to the economic sustainability of public banks. We found that a swift transition from strategy A to C can play a vital role in preventing public banks worldwide from bankrupting. We also found that a pre-freezing level of 18 x 10^8^ TNC would be a cost-effective strategy to deliver therapeutic value to patients with a minimum financial deficit for the bank. In a context of limited public spending on health systems, banking decisions based on stronger selection criteria are essential if public banks are to remain financially sustainable and maximize their long-term therapeutic value for patients.

## Supporting Information

S1 Table Distribution of CBUs. (PDF)

S2 Table Characteristics of CBUs. (XLSX)
