# Supplementary material for: Banking or Bankrupting: Strategies for Sustaining the Economic Future of Public Cord Blood Banks
Source: PLoS One. 2015 Dec 1;10(12):e0143440. doi: 10.1371/journal.pone.0143440 (PMC4666404; doi:10.1371/journal.pone.0143440)
Supplement: S1 Table — (PDF) [file pone.0143440.s001.pdf]

S1 TABLE: DISTRIBUTION OF CBUs

	TOTAL		RELEASED nationally			RELEASED internationally		
	Caucasian	Non Caucasian	Caucasian	Non Caucasian	Total	Caucasian	Non Caucasian	Total
DRESDEN (n=4,074)	3, 235	839	0	0	0 (0.0%)	20	5	25 (100.0%)
DUKE (n=3,750)	2, 067	1, 683	100	64	164 (77.7%)	28	19	47 (22.3%)
St LOUIS (n=1,397)	663	734	11	12	23 (53.5%)	8	12	20 (46.5%)
MARSEILLE (n=175)	64	111	1	2	3 (60.0%)	0	2	2 (40.0%)
